# The Investigation into the Toxic Potential of Iron Oxide Nanoparticles Utilizing Rat Pheochromocytoma and Human Neural Stem Cells

**DOI:** 10.3390/nano9030453

**Published:** 2019-03-18

**Authors:** Weili Ma, Paul M. Gehret, Richard E. Hoff, Liam P. Kelly, Won Hyuk Suh

**Affiliations:** Department of Bioengineering, College of Engineering, Temple University, Philadelphia, PA 19122, USA; weili.ma@temple.edu (W.M.); tuf82330@temple.edu (P.M.G.); tuf34380@temple.edu (R.E.H.); tuf67109@temple.edu (L.P.K.)

**Keywords:** nanoparticles, magnetite, Fe_3_O_4_, hydrothermal synthesis, surface functionalization, stem cells, neuronal differentiation, biocompatibility, neurotoxicity, cytotoxicity

## Abstract

Magnetic iron oxide (Magnetite, Fe_3_O_4_) nanoparticles are widely utilized in magnetic resonance imaging (MRI) and drug delivery applications due to their superparamagnetism. Surface coatings are often employed to change the properties of the magnetite nanoparticles or to modulate their biological responses. In this study, magnetite nanoparticles were fabricated through hydrothermal synthesis. Hydrophobicity is often increased by surface modification with oleic acid. In this study, however, hydrophobicity was introduced through surface modification with n-octyltriethoxysilane. Both the uncoated (hydrophilic) and coated (hydrophobic) individual nanoparticle sizes measured below 20 nm in diameter, a size range in which magnetite nanoparticles exhibit superparamagnetism. Both types of nanoparticles formed aggregates which were characterized by SEM, TEM, and dynamic light scattering (DLS). The coating process significantly increased both individual particle diameter and aggregate sizes. We tested the neurotoxicity of newly synthesized nanoparticles with two mammalian cell lines, PC12 (rat pheochromocytoma) and ReNcell VM (human neural stem cells). Significant differences were observed in cytotoxicity profiles, which suggests that the cell type (rodent versus human) or the presence of serum matters for nanoparticle toxicology studies. Differences in nanoparticle associations/uptake between the two cell types were observed with Prussian Blue staining. Finally, safe concentrations which did not significantly affect neuronal differentiation profiles were identified for further development of the nanoparticles.

## 1. Introduction

Magnetic nanoparticles (NPs), usually made from iron (II, III) oxide (magnetite, Fe_3_O_4_), are widely utilized in various biomedical applications such as targeted drug delivery [1,2] and as contrast agents in magnetic resonance imaging (MRI) [3,4,5]. Materials that are ferromagnetic and ferrimagnetic possess spontaneous magnetic domains, which are regions within the materials where the individual atom’s magnetic moments are aligned. At a small enough size, only a single domain will be formed, leading to strong magnetization within a magnetic field. This phenomenon, called superparamagnetism, occurs in nanoparticles of a certain size. The size required to induce superparamagnetism is material-dependent, but is frequently found within the range of 10–20 nm [6]. A notable property of superparamagnetic materials is that they are unable to retain their magnetization when the magnetic field is removed. This lack of magnetic memory allows the nanoparticles to not aggregate due to magnetic polarity, allowing them to maintain their colloidal stability and avoid any embolization of the blood vessels when they are utilized in vivo [6,7].

Initial investigations on superparamagnetic NPs have limited reports on biological responses, but rather focused on synthesis routes and characterization of the physicochemical properties [7]. Crystallinity, size control, and shape are among the important considerations for selecting a process for the chemical synthesis of magnetic nanoparticles [8,9]. This study uses hydrothermal synthesis, which produces particles through the process of subjecting a high temperature aqueous solution to high vapor pressure [10]. The size of the resulting NPs produced by this method can be controlled by reactant concentration, reaction time, and temperature [11,12]. Compared to other methods, such as chemical and biosynthesis of magnetite [13,14,15], hydrothermal synthesis can be performed as a one-pot reaction. In addition, using ethylene glycol as the solvent alleviates the oxidation of Fe^2+^, allowing the reaction to proceed in the presence of oxygen [16]. When coated with various functional groups, Fe_3_O_4_ NPs can present differences in cytotoxicity and genotoxicity to different cell types [17]. In addition, when injected in vivo, protein coronas are more likely to form on TiO_2_ and Au-coated magnetite NPs compared to polyvinyl alcohol (PVA) and SiO_2_-coated NPs [18]. In this study, we investigate the changes to cytotoxicity in vitro after surface coating with n-octyltriethoxysilane. The use of silane-based coatings for magnetite nanoparticles has been reported to enhance biocompatibility [19].

**Figure 1:**
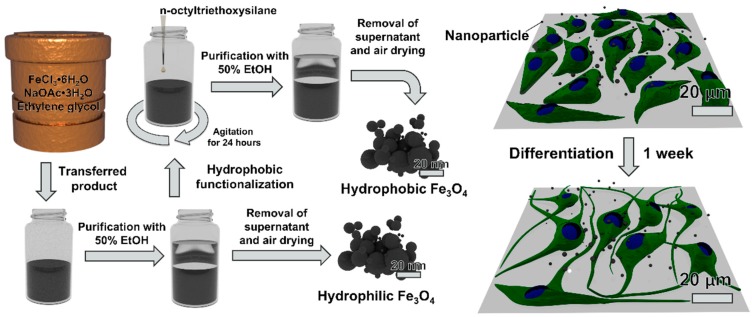
Schematic flow chart for the synthesis of magnetite nanoparticles and biological testing.

The toxic effects of magnetic NPs, especially on neural-type cells, have been of strong interest due to increased use as MRI contrast agents [20,21,22,23]. Moreover, these NPs have been reported to possess the ability to cross the blood-brain barrier (BBB) and directly reach the brain tissue [24,25]. In the literature, neurotoxicity of magnetic NPs has been evaluated utilizing a variety of neural-type cells including neuronal cancer and neural progenitor cells, from non-human mammals and humans [26,27,28,29,30]. Ultimately, while a certain degree of neurotoxicity has been observed from these nanoparticles, it is difficult to compare existing biological results between studies due to differences in NP synthesis conditions, surface modifications, and cell types. As seen in Figure 1, the main objective of this study was to evaluate the neurotoxicity of magnetite NPs synthesized via hydrothermal synthesis with PC12 rat pheochromocytoma and ReNcell VM, an immortalized human neural stem cell line [31]. Compared to neural stem cells, the PC12 cell line is an inexpensive model for neuronal studies and has been utilized for nanoparticle toxicology studies [32]. Additionally, we evaluate how biological results are affected when iron oxide nanomaterials are coated with n-octyltriethoxysilane, which results in hydrophobic surfaces.

## 2. Materials and Methods

Hydrochloric acid (HCl, A144-500), ferric chloride hexahydrate (I88-100), Hoechst 33342 (62249), PrestoBlue™ cell viability reagent (A13261), LIVE/DEAD viability/cytotoxicity kit (L3224), and donkey anti-rabbit IgG Alexa Fluor 488 (A-21206) were purchased from Thermo Fisher Scientific (Waltham, MA, USA)**.** Potassium ferricyanide (20150) was purchased from Electron Microscopy Sciences (Hatfield, PA, USA)**.** Aluminum potassium sulfate dodecahydrate (36288) was purchased from Alfa Aesar (Haverhill, MA, USA). Ethylene glycol (044710) was purchased from Oakwood Chemical (Estill, SC, USA). Ethanol (2701) was purchased from Decon Laboratories, Inc. (King of Prussia, PA, USA). Glacial acetic acid (423220025), dichloromethane (D37-500), n-octyltriethoxysilane (338080250), Nuclear Fast Red (211980010), and paraformaldehyde (PFA, 416785000) were purchased from Acros Organics (part of Thermo Fisher Scientific, Waltham, MA, USA). Deionized water ASTM Type II (9765-10L) was purchased from Aqua Solutions (Deer Park, TX, USA). RPMI 1640 with l-glutamine (10040CV), Trypsin-EDTA (25-053-CI), type I rat tail collagen (354236), and 100 × penicillin-streptomycin were purchased from Corning, Inc. (Corning, NY, USA). ReNcell VM (RVM, SCC008), EmbryoMax^®^ Dulbecco’s phosphate buffered saline (PBS) without calcium and magnesium (BSS-1006-B), ReNcell Maintenance Medium (RMM, SCM005), basic fibroblast growth factor (bFGF, GF003), epidermal growth factor (GF144), Accutase (SCR005), Dulbecco’s modified Eagle’s medium with Ham’s F12 supplement (DMEM/F12, DF-041-B), laminin (CC095), β-III tubulin antibody (AB9354), growth associated protein-43 antibody (GAP-43, AB5220), and FITC-conjugated donkey anti-chicken IgY secondary antibody (AP194F) were purchased from Millipore Sigma (Burlington, MA, USA). Type I rat tail collagen was purchased from BD Biosciences (San Jose, CA, USA). BenchMark™ fetal bovine serum (100–106) was purchased from Gemini Bio-Products (Sacramento, CA, USA). HyClone™ heat-inactivated horse serum (SH30074.02HI) was purchased from GE Life Sciences (Pittsburgh, PA, USA). Donkey serum (D9663), Triton™ X-100 (X100), nerve growth factor beta (N2513), sodium acetate trihydrate (S7670-250G), and laminin (L2020) were purchased from Sigma-Aldrich (St. Louis, MO, USA). Donkey anti-rabbit IgG (H + L) Alexa Fluor 488 secondary antibody and cell culture plastics including BioLite 96-well plates and 25 (or 75) cm^2^ flasks were purchased from Thermo Fisher Scientific (Waltham, MA, USA)**.** The PC12 cell line was kindly provided by Temple University’s Department of Bioengineering (Philadelphia, PA, USA).

### 2.1. Hydrothermal Synthesis of Magnetite Nanoparticles

Ferric chloride hexahydrate (405 mg, 1.50 mmol) and sodium acetate trihydrate (1.08 g, 7.9 mmol) were added to an autoclave vessel (Parr Instrument Company, Moline, IL, USA) along with ethylene glycol (15 mL, 269 mmol). The autoclave vessel was assembled and placed into a furnace (Barnstead Thermolyne FB1415M, Waltham, MA, USA) at 200 °C for 24 h. After 24 h had passed, the vessel was removed from the furnace with tongs and allowed to cool to room temperature. Excess ethylene glycol was removed, and the nanoparticles were transferred to a 50 mL conical tube with 20 mL of 50% (v/v) ethanol in water. The nanoparticles were dispersed by ultrasonication at 185 W for 5 min (Fisher Scientific FS110D), followed by pelletization via centrifugation at 1000 × *g* for 5 min. A magnet was used to pull the smaller magnetic particles to the bottom of the tube. The supernatant liquid was removed, and the nanoparticles were washed with 20 mL of 50% (v/v) ethanol in water two more times. After the final wash, nanoparticles were air dried in a chemical fume hood. These non-functionalized nanoparticles were designated as hydrophilic nanoparticles.

### 2.2. Hydrophobic Functionalization of Magnetite Nanoparticles

For surface functionalization, the nanoparticles were dispersed in 20 mL of 50% (v/v) ethanol in water prior to air drying. The coating process utilized to surface modify the nanoparticles with n-octyltriethoxysilane is based on the Stöber method [33]. The samples were placed on an orbital shaker (Fisherbrand Incubating Mini-Shaker 02217753) fitted with a 50 mL tube holder. While the sample was shaking (400 rpm, room temperature), n-octyltriethoxysilane (60 µL, 0.191 mmol) was added. The sample was left to shake for 24 h. The functionalized nanoparticles were washed and dried following the protocol above for non-functionalized nanoparticles. The functionalized nanoparticles were designated as hydrophobic nanoparticles.

### 2.3. Electron Microscopy and Nanoparticle Sizing

For scanning electron microscopy (SEM), dried hydrophilic and hydrophobic nanoparticles were attached to a piece of glass slide using double-sided carbon tape and further dried under vacuum for 1 h. The glass slide was then mounted on an SEM stub using copper tape and sputter coated with gold. An Agilent 8500 FE-SEM was used to obtain SEM images.

For transmission electron microscopy (TEM), dried hydrophilic and hydrophobic nanoparticles were suspended at a concentration of 100 µg/mL (in 50/50% v/v ethanol/water) and 2 µL of this suspension was added to a TEM grid. The sample was air dried overnight and then imaged with a JEOL JEM 1400 TEM microscope fitted with a GATAN UltraScan 1000 CCD camera. The ImageJ software [34] was used to size the diameters of individual nanoparticles from TEM images and aggregate sizes were measured from SEM images. In total, 240 individual nanoparticles were sized across 8 TEM images for both the hydrophilic and hydrophobic nanoparticles. For aggregate sizing, over 50 aggregates were sized from across 5 SEM images for both hydrophilic and hydrophobic nanoparticles.

### 2.4. Dynamic Light Scattering and ζ-Potential

Dynamic light scattering (DLS) and ζ-potential (zeta potential) measurements were conducted on a Zetasizer Nano ZS (Malvern Panalytical, Malvern, Worcestershire, UK) to assess the hydrodynamic radii and colloidal stability of nanoparticles, respectively, in aqueous media (pH 7.0). Nanoparticles were prepared at 0.1 mg/mL concentrations, diluted from a 10 mg/mL stock suspension. Immediately before measurements, the nanoparticle suspensions were ultrasonicated at 185 W for 5 min (Fisher Scientific FS110D). Following ultrasonication, the nanoparticle suspensions were transferred into 1 mL disposable cuvettes with 1 cm path length for DLS and disposable folded capillary cells (DTS1060) for ζ-potential measurements.

### 2.5. X-Ray Diffraction

Hydrophilic and hydrophobic nanoparticles samples were probed for Fe_3_O_4_ crystal structure using powder X-ray diffraction. Samples were ground into fine powder using a glass tissue grinder in a 50 mL conical tube. Spectra was obtained using a Bruker D8 Advance instrument (Billerica, MA, USA) fitted with a copper X-ray source and a LYNXEYE XE detector. Scattering was attenuated with an anti-scatter screen and a nickel foil filter to attenuate Kβ radiation. The spectra were obtained using DIFFRAC.SUITE software and post-analysis was done using DIFFRAC.EVA software.

### 2.6. Nanoparticle Sterilization

Dried nanoparticles were weighed and suspended in 70% (v/v) ethanol in water. Nanoparticles were dispersed by ultrasonication at 185 W (Fisher Scientific FS110D) for 5 min, followed by vortexing for 15 s. The particles were then centrifuged at 7000 × *g* for 10 min and decanted in a sterile biosafety cabinet. This was repeated a total of three times. Afterwards, the nanoparticles were washed three times with sterile deionized water using the same protocol. After the final water wash, the nanoparticles were dispersed at a final concentration of 10 mg/mL in sterile deionized water.

### 2.7. Cell Culture

PC12 rat pheochromocytoma cells from adrenal medulla were maintained in RPMI 1640 medium supplemented with 10% heat-inactivated horse serum, 5% fetal bovine serum, and 1% penicillin-streptomycin (PC12 proliferation medium). The cells were cultured at standard incubation conditions (5% CO_2_ at 37 °C). Cells were passaged at 70% confluency by incubating the cells with Trypsin-EDTA. Cells were pelletized by centrifugation at 200 × *g* and the media were changed every 24 to 48 h. Tissue culture plastic for PC12 culture was collagen-coated prior to cell seeding. Type I rat tail collagen was diluted to 50 µg/mL in 0.02 M acetic acid, which was filter sterilized through a 0.22 µm membrane. The collagen solution was incubated for at least 2 h at 37 °C. After removing the collagen solution, the tissue culture plastic was rinsed twice with PBS.

ReNcell VM (RVM) human stem cells were cultured in ReNcell maintenance media supplemented with 20 ng/mL EGF, 20 ng/mL bFGF, and 1% penicillin-streptomycin (RVM proliferation medium). Cell culture medium was changed every 24 to 48 h. RVMs were passaged when the flask reached 70% confluency by treating the cells with Accutase at room temperature for 5 min. Cells were pelletized by centrifugation at 200 × *g* for 5 min. Tissue culture plastic for RVM culture was laminin-coated prior to cell seeding. The laminin was diluted to 20 µg/mL in DMEM/F12 medium and incubated for at least 4 h at 37 °C. After removing the laminin solution, the tissue culture plastic was air dried for 5 min.

### 2.8. Cell Viability and LIVE/DEAD Staining

PC12 and RVM cells were seeded in 96-well plates at a density of 1 × 10^4^ cells per well with 100 µL proliferation media. The cells were stabilized for 16 to 24 h and media changed prior to treatment with varying amounts of nanoparticles. After sonication, the sterile 10 mg/mL nanoparticle stock suspension was diluted to 1 mg/mL with sterile deionized water. Starting at 64 µL (64 µg nanoparticles, corresponding to approximately 0.39 mg/mL), serial dilutions were performed using sterile deionized water (1:2 per dilution down to 0.5 µg nanoparticles, corresponding to approximately 0.003 mg/mL). To each well containing 100 µL proliferation medium, 64 µL of diluted nanoparticle suspensions were added directly (total final volume 164 µL per well). Sterile deionized water (64 µL) without nanoparticles was added to control wells. After 24 h, cells were washed three times with PBS. For quantitative viability analysis, the cells were treated with PrestoBlue™ cell viability reagent following the manufacturer’s protocol. For LIVE/DEAD staining, 10 µL LIVE/DEAD solution was added to each well without washing the cells (final concentrations: 2 µM Calcein AM and 4 µM Ethidium Homodimer-1). After incubation for 30 min, the cells were washed three times with proliferation medium and imaged with an Olympus IX83-DSU microscope equipped with a Hamamatsu ORCA-R2 CCD camera and a Chamlide LCI live cell imaging stage-top incubator.

### 2.9. PC12 and RVM Differentiation with Nanoparticles 

PC12 cells and RVMs were seeded onto 96-well plates at a density of 1 × 10^4^ cells per well with 100 µL proliferation media. After stabilizing for 16 to 24 h, cells were treated with 1 µg hydrophilic or hydrophobic nanoparticles, corresponding to a final concentration of approximately 0.006 mg/mL (164 µL total well volume). After 24 h, nanoparticle-containing medium was removed, and all wells were washed three times with proliferation media. PC12 cells were differentiated by treating with 50 ng/mL NGF diluted in RPMI 1640 supplemented with 1% heat-inactivated horse serum and 1% penicillin-streptomycin (PC12 differentiation medium). RVM cells underwent spontaneous differentiation upon withdrawal of growth factors, EGF and bFGF, from the proliferation medium (RVM differentiation medium). The differentiation medium was refreshed every 48 h. The cells were differentiated for 7 days and then fixed by treating with 4% (w/v) PFA for 10 min. The cells were washed with PBS three times and were stored at 4 °C in PBS until antibody staining.

### 2.10. Immunocytochemistry

Fixed cells were permeabilized and blocked with a solution of 5% (v/v) donkey serum and 0.3% (v/v) Triton™ X-100 in PBS for 30 min at room temperature. The samples were then washed three times with PBS. Primary antibodies (β-III tubulin; AB9354, GAP-43; AB5220) were diluted 1:200 in blocking solution and samples were incubated for 2 h at room temperature or overnight at 4 °C. Samples were then washed again with the protocol above, followed by incubation with 1:200 diluted fluorophore-conjugated secondary antibodies for 1 h at room temperature. After another PBS wash, nuclei were stained with 1 µg/mL Hoechst 33342 in PBS for 15 min at room temperature. The samples were washed a final time with PBS and were kept hydrated in fresh PBS for imaging on an Olympus IX83-DSU microscope equipped with a Hamamatsu ORCA-R2 CCD camera. 

ImageJ was used to quantify the expression of β-III tubulin or growth associated protein 43 (GAP-43) from immunocytochemistry samples. The channels for each image were separated and the nuclei were counted using an in-house programmed ImageJ macro for consistency. Briefly, the nuclei channel was background subtracted and contrast enhanced, followed by conversion to binary. The resulting image was analyzed with the particle analysis plug-in with watershed enabled. The β-III tubulin and GAP-43 fluorescence was background corrected by first duplicating the channel. A 200-pixel Gaussian blur was applied to the copy, which was then subtracted from the original image. The resulting image was auto contrast enhanced and merged with the blue nuclei channel. The number of cells expressing β-III tubulin or GAP-43 was manually counted using the cell counter plug-in. Total positive expression was calculated from the ratio of β-III tubulin or GAP-43 positive cells to total nuclei in each individual image.

### 2.11. Prussian Blue and Nuclear Fast Red Staining

Prussian blue staining was carried out on fixed, differentiated cells using a previously published procedure by Miyoshi et al. [35]. Identical to the differentiation protocol, cells were treated with 1 µg nanoparticles for 24 h prior to differentiation. This amount of nanoparticles corresponds to approximately 0.006 mg/mL (164 µL total well volume). A solution of 4% (w/v) potassium ferricyanide and 4% (v/v) HCl in water were separately prepared by dissolving overnight at room temperature using a magnetic stirrer. These solutions were then combined at a 1:1 ratio and added to differentiated, fixed PC12 and RVM cells. The samples were incubated at 37 °C for 20 min and then washed three times with PBS. A solution of 0.1% (w/v) nuclear fast red and 5% (w/v) aluminum sulfate was added to each well. The samples were incubated for 20 min, followed by sequential washing with PBS three times and deionized water three times. Images were obtained using an Olympus CKX-41 microscope equipped with an Infinity1-2CB camera. Using the Prussian blue and nuclear fast red images, the number of positively stained cells was determined. Positively stained cells were defined as cells with the Prussian blue stain overlapping the cell body.

### 2.12. Statistical Analysis

Data analysis was performed using JMP, MATLAB, and Microsoft Excel. A Student’s *t* test with an α = 0.05 was performed to compare nanoparticle sizes. For cytotoxicity experiments, treatment groups were compared by one-way ANOVA and Dunnett’s test against the control group. A p-value less than 0.05 was considered statistically significant. To compare hydrophilic and hydrophobic particles at the same treatment condition, a Student’s *t* test with an α = 0.05 was performed.

## 3. Results

**Figure 2:**
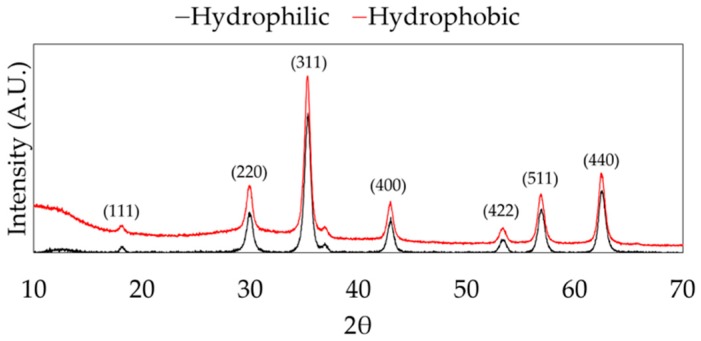
X-ray diffraction spectra of hydrophilic and hydrophobic nanoparticles. The peaks in both results matched with crystallography open database peaks associated with magnetite (Fe_3_O_4_).

Magnetite nanoparticles were successfully synthesized using the hydrothermal method and surface functionalized with n-octyltriethoxysilane. The XRD spectra show representative peaks for magnetite (Fe_3_O_4_) in both hydrophilic and hydrophobic nanoparticle samples, as shown in Figure 2.

**Figure 3:**
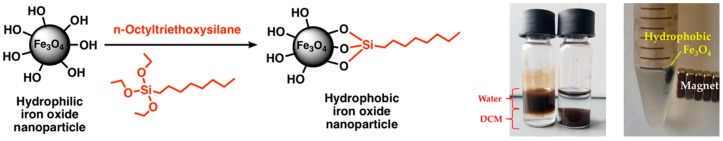
Surface functionalization of iron oxide nanoparticles using n-octyltriethoxysilane. (First photograph) Hydrophobic magnetite nanoparticles (NPs) phase separate into organic medium. Left: hydrophilic nanoparticles. Right: hydrophobic nanoparticles. (Second photograph) Hydrophobic Fe_3_O_4_ attracted by a set of magnets.

The functionalization with n-octyltriethoxysilane increased the hydrophobicity of the magnetite nanoparticles. In a mixture of 50/50% (v/v) DCM/water, the hydrophobic nanoparticles settle in the organic phase, while the hydrophilic nanoparticles remain in the aqueous phase, as shown in Figure 3. Furthermore, the hydrophobic nanoparticles retain their magnetic properties.

**Figure 4:**
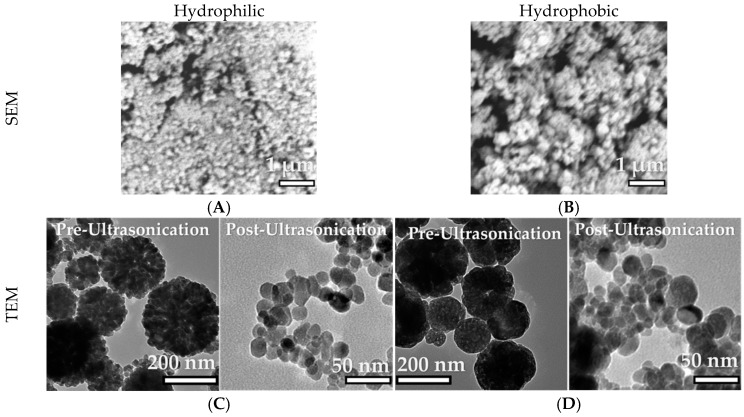

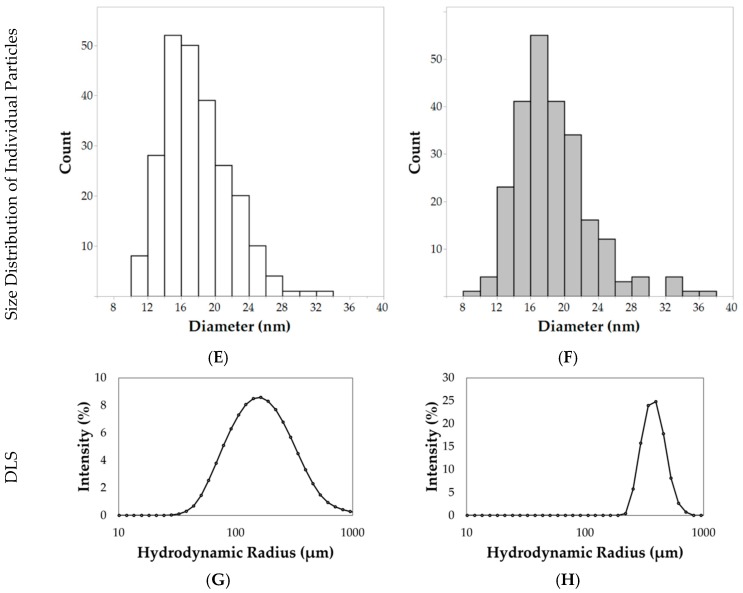
Electron microscopy images and size distribution histograms for the synthesized iron oxide nanoparticles. SEM images of (**A**) hydrophilic and (**B**) hydrophobic nanoparticles agglomerations 35,000× with a scale bar of 1 µm. TEM images show agglomerated (left, 30,000×, scale bar = 200 nm) and individual (right, 100,000×, scale bar = 50 nm) (**C**) hydrophilic and (**D**) hydrophobic nanoparticles. Agglomerated nanoparticles are observed if the sample is not sonicated. After sonication, the majority of nanoparticles are dispersed. A statistically significant difference was observed in the size distributions of individual (**E**) hydrophilic and (**F**) hydrophobic nanoparticles. Dynamic light scattering (DLS) results show the aggregation states of (**G**) hydrophilic and (**H**) hydrophobic nanoparticles.

Electron micrographs of the nanoparticles were acquired for size analysis, as shown in Figure 4A–D. Diameters of individual nanoparticles dispersed by ultrasonication were measured using the TEM images, as shown in Figure 4C,D (TEM analysis). The hydrophilic nanoparticles synthesized using the 24 h hydrothermal synthesis method resulted in an average diameter (±standard deviation) of 17.9 ± 3.9 nm, as shown in Figure 4E. After hydrophobic coating with n-octyltriethoxysilane, nanoparticles had an average diameter of 18.7 ± 4.4 nm, as shown in Figure 4F. Although the average diameters were very similar, the size of individual nanoparticles, as shown in Figure 4C,D after the n-octyltriethoxysilane surface coating, were found to have a statistically significant increase in diameter (Student’s *t* test, *p* < 0.05, directional test, N = 240 per sample).

Aggregations of nanoparticles were observed by low magnification SEM analysis, as shown in Figure 4A,B (SEM analysis). Hydrophilic nanoparticle aggregates measured 164.6 ± 33.4 nm in diameter. After the surface passivation of a hydrophobic component (n-octyltriethoxysilane), the aggregate size increased to 221.5 ± 52.0 nm, as shown in Figure 4B (SEM analysis). These results were confirmed with TEM analysis of aggregates at lower magnification, as shown in Figure 4C,D (TEM analysis). This increase was statistically significant when analyzed via a Student’s *t* test (N = 52 per sample, *p* < 0.0001). DLS measurements at 0.1 mg/mL in deionized water indicate formations of nanoparticle aggregates, as shown in Figure 4G,H. The hydrophilic nanoparticles had a Z-average of 154.9 ± 1.5 nm with a PDI (polydispersity index) of 0.35. In comparison, hydrophobic nanoparticles had a Z-average of 594.1 ± 36.5 nm with a PDI of 0.47. The ζ-potential for hydrophilic nanoparticles measured −16.1 ± 5.4 mV while the hydrophobic nanoparticles measured −27.7 ± 8.2 mV.

**Figure 5:**
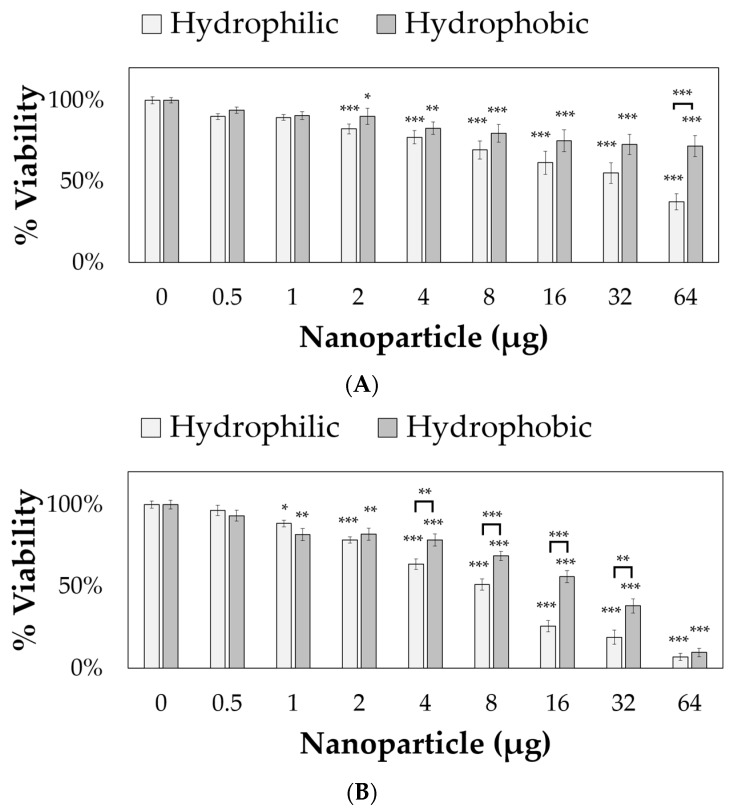
Hydrophilic and hydrophobic nanoparticle cytotoxicity after 24 h incubation with (**A**) PC12 cells and (**B**) ReNcell VM (RVM) cells. Error bars depicted as standard error. * *p* < 0.05, ** *p* < 0.01, *** *p* < 0.001.

The nanoparticles were applied to 10,000 RVM and PC12 cells for 24 h, and cell viabilities were measured using PrestoBlue™, as shown in Figure 5. Both hydrophilic and hydrophobic nanoparticles were toxic to PC12 cells starting at 2 µg (per 10,000 cells and 164 µL total volume, corresponding to approximately 0.012 mg/mL) resulting in 82.5% and 90.1% viability values, respectively. For RVM cells, significant cytotoxicity was observed at 1 µg (corresponding to approximately 0.006 mg/mL) with only 88.5% and 81.8% viability values after treatment with hydrophilic and hydrophobic nanoparticles, respectively. For both cell lines, the hydrophobic coating attenuated cytotoxic effects at various concentrations. In PC12 cells, 64 µg of hydrophobic nanoparticles (corresponding to approximately 0.39 mg/mL) resulted in 71.8% viability values while the hydrophilic nanoparticles only showed 37.5% viability. The reduced cytotoxicity from the hydrophobic coating was also observed in 4, 8, 16, and 32 µg nanoparticle treatment groups (corresponding to approximately 0.024, 0.049, 0.098, and 0.195 mg/mL, respectively) for RVM cells. At 64 µg (corresponding to approximately 0.39 mg/mL), this effect was not observed in RVM cells due to low viabilities at 7.0% and 9.7% for hydrophilic and hydrophobic nanoparticle treatment groups, respectively.

**Figure 6:**
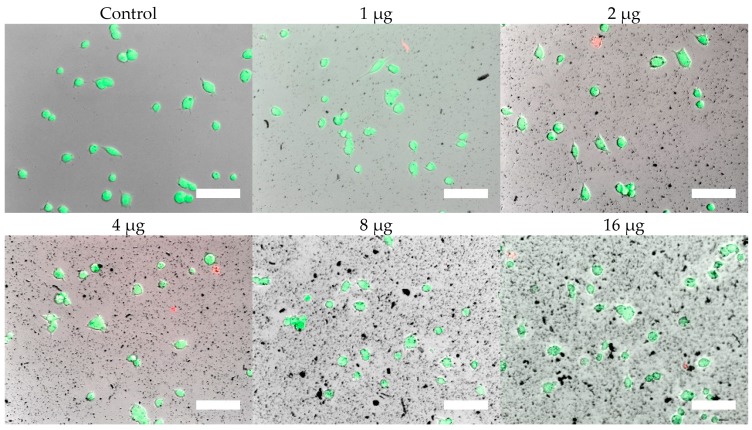
LIVE/DEAD assay results of PC12 cells treated with hydrophilic iron oxide nanoparticles after 24 h. Calcein (green) indicates live cells while ethidium homodimer-1 (red) indicates dead cells. Scale bar = 50 µm.

**Figure 7:**
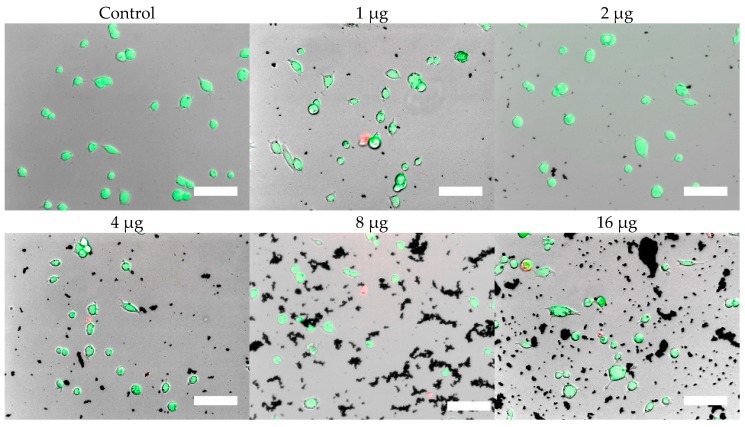
LIVE/DEAD assay results of PC12 cells treated with hydrophobic iron oxide nanoparticles after 24 h. Calcein (green) indicates live cells while ethidium homodimer-1 (red) indicates dead cells. Scale bar = 50 µm.

**Figure 8:**
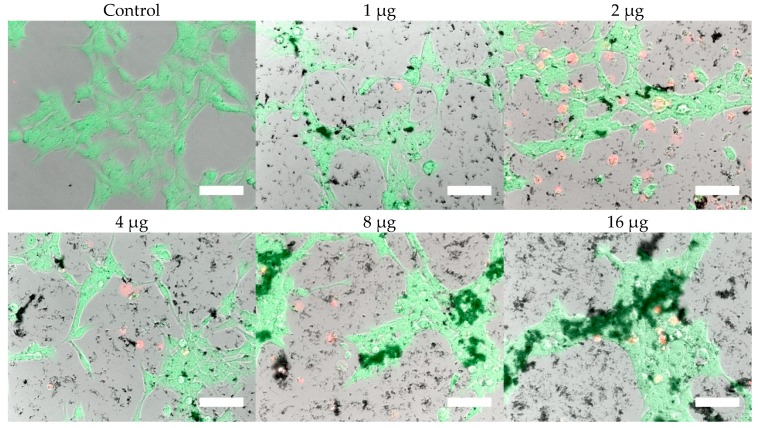
LIVE/DEAD assay results of RVM cells treated with hydrophilic iron oxide nanoparticles after 24 h. Calcein (green) indicates live cells while ethidium homodimer-1 (red) indicates dead cells. Scale bar = 50 µm.

**Figure 9:**
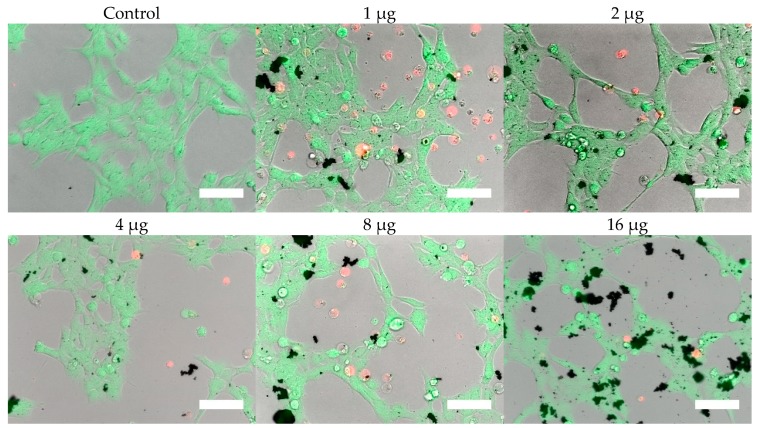
LIVE/DEAD assay results of RVM cells treated with hydrophobic iron oxide nanoparticles after 24 h. Calcein (green) indicates live cells while ethidium homodimer-1 (red) indicates dead cells. Scale bar = 50 µm.

Cells were, in addition, stained with the LIVE/DEAD assay for live-cell fluorescence imaging, as shown in Figure 6, Figure 7, Figure 8 and Figure 9. At 0.5 µg (not shown, corresponding to approximately 0.003 mg/mL), no discernible changes to cell viability were detected and morphological differences were not observed. Some changes in cell morphology occur as nanoparticle amount is increased from 1 to 16 µg (corresponding to 0.006 and 0.098 mg/mL, respectively). There were more rounded cells and less neurites were observed in the RVM cells. With PC12 cells, the normal morphology is more rounded, so it is difficult to assess morphological changes. The 32 and 64 µg treatments (not shown, corresponding to 0.195 and 0.39 mg/mL, respectively) formed dense aggregations in the medium which did not allow for a clear visualization of cell morphology. LIVE/DEAD fluorescence results indicate large quantities of dead cells with increasing nanoparticle amount. The hydrophobic nanoparticles were also seen to form denser aggregates compared to hydrophilic nanoparticles, corresponding to SEM and DLS aggregate size analysis results.

**Figure 10:**
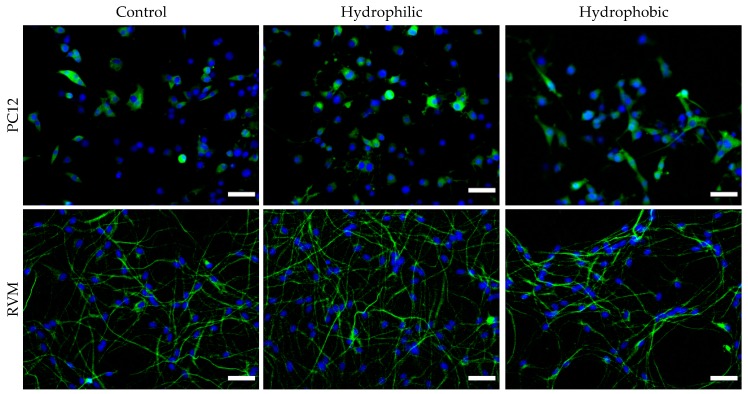
Immunocytochemistry of PC12 and RVM differentiated for 1 week after 24 h treatment with nanoparticles. PC12 cells were stained for GAP-43, a neuronal growth cone marker and RVM were stained for β-III tubulin, a neuronal differentiation marker. No significant differences were observed between the treatment and control groups. N ≥ 1,100 cells counted per condition and cell line. Scale bar = 50 µm.

To further investigate the neurotoxic effects of the nanoparticles, neuronal differentiation was carried out after exposing the cells to nanoparticles for 24 h. After 7 days in differentiation medium, PC12 cells were stained for GAP-43 (neuronal growth cone marker) and RVM cells were stained for β-III tubulin (early neuronal differentiation marker). Fluorescence images were obtained, and the number of positively-stained cells were manually counted in ImageJ, as shown in Figure 10. After counting more than 1,100 cells per condition over multiple plates and images, it was determined that 24 h treatment with 1 µg nanoparticles (corresponding to 0.006 mg/mL) presented no statistical significance in differentiation characteristics compared to control group cells (for each cell line). 

**Figure 11:**
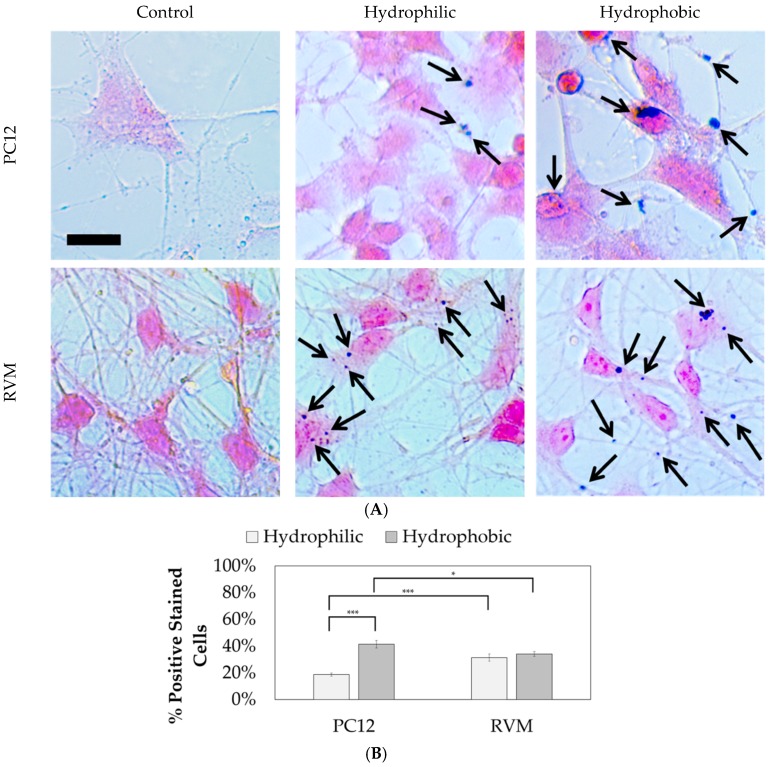
Brightfield microscopy images of PC12 and RVM cells after 24 h of nanoparticle treatment followed by 7 day differentiation. (**A**) Prussian blue visualizes the remaining iron oxide and nuclear fast red is used as the counterstain. Black arrows indicate iron oxide particles associated with the cells. Scale bar = 10 µm. (**B**) Prussian blue positive cell count analysis results. N ≥ 460 cells over 25 images per condition. * *p* < 0.05, *** *p* < 0.001.

Prussian blue staining was performed to locate the iron oxide nanoparticles relative to the cells after differentiation, as shown in Figure 11. Nuclear fast red was used as a counterstain. The hydrophilic nanoparticles were observed in 18.7 ± 6.2% of PC12 cells compared to 41.5 ± 16.7% for hydrophobic nanoparticles. On the other hand, no statistical significance was observed between hydrophilic and hydrophobic particles for RVM cells which resulted in 31.4 ± 14.0% and 34.0 ± 9.7% associations, respectively. When comparing the two cell types, however, the RVM cells had a significantly increased association of hydrophilic nanoparticles while PC12 cells had a significantly higher association of hydrophobic nanoparticles. These results suggest that iron oxide nanoparticles are, in fact, interfaced to the mammalian cells that underwent cellular differentiation, but we cannot necessarily distinguish whether the nanoparticles have been internalized or not. The goal of this study was to treat the cells with the synthetic iron oxide nanoparticles and determine if neuronal differentiation was affected. Based on the immunofluorescence staining for differentiation markers as shown in Figure 10, iron oxide nanoparticles at 1 µg per 10,000 cells (regardless of their hydrophilicity or hydrophobicity) did not alter the differentiation profiles for PC12 and RVM cells (no neurotoxicity is observed at this concentration).

## 4. Discussion

**Scheme 1:**
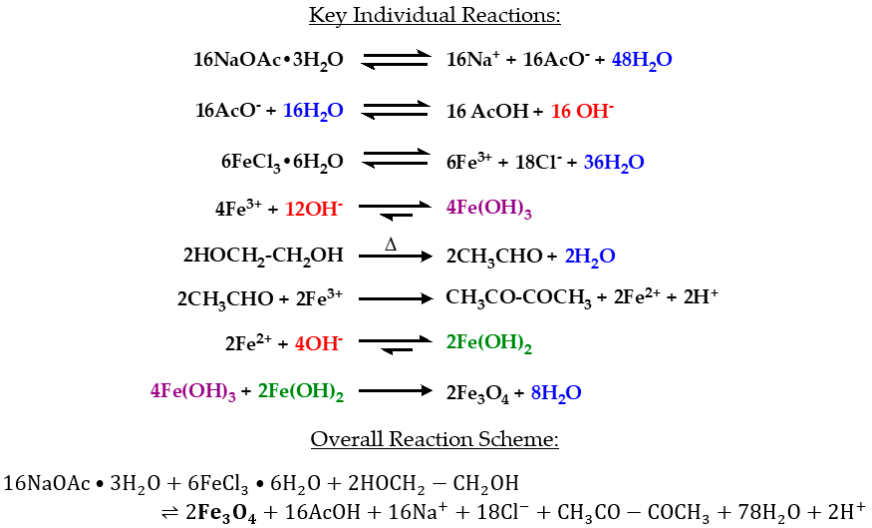
The reactions involved in the production of iron oxide (magnetite) nanoparticles.

Hydrothermal synthesis has been utilized for synthesizing magnetite particles [10]. A source of Fe^3+^ ions, such as ferric chloride in water, is heated in the presence of ethylene glycol, which acts as a reducing agent to produce Fe^2+^ ions [36,37]. Both Fe^2+^ and Fe^3+^ ions must be present because both ions are within the structure of iron (II, III) oxide. An electrostatic stabilizer such as sodium acetate is also needed to prevent particle agglomeration during synthesis [10,38]. The method presented in this paper involves hydrated forms of ferric chloride and sodium acetate in ethylene glycol. The reaction mixture is placed within an autoclave vessel and nanocrystals form as the vessel is heated to 200 °C and kept constant for 24 h [38]. This process produces iron oxide (magnetite) nanoparticles through the following series of reactions (Scheme 1) [39]:

A critical reaction step is the formation of acetaldehyde by the dehydration of ethylene glycol in a pinacol rearrangement [40]. Acetaldehyde is most commonly known as a byproduct of alcohol metabolism in the body. In excess, acetaldehyde has been suggested to be carcinogenic [41,42]. The major byproduct of this reaction, however, has been reported to be diacetyl which forms after the reduction of Fe(III) to Fe(II) by acetaldehyde [43]. Recently, exposure to acetyls has been linked to the development of obliterative bronchiolitis [44]. Although the presence of acetaldehyde and acetyls were not tested in this study, care should be taken during synthesis to avoid exposure. The autoclave vessel must be allowed to cool to room temperature, and reaction products should be handled in a chemical fume hood with proper personal protective equipment.

The size of the individual magnetite nanoparticles is of great importance since superparamagnetism occurs in a specific size range, as previously stated in the introduction. Using the hydrothermal synthesis method, magnetite nanoparticles could be prepared in a size range with superparamagnetic properties. The size of the nanoparticles also play a vital role in uptake by cells, with larger nanoparticles generally exhibiting reduced uptake [45]. Aggregations (such as the ones seen in Figure 4) can affect biological results such as cytotoxicity and stem cell differentiation and must be taken into consideration when interpreting these results [46,47]. We observed that the bulk of the as-prepared nanoparticles were agglomerates as seen in Figure 4, which was confirmed by SEM, TEM, and DLS. Such agglomerated iron oxide nanoparticles have been reported by others [36,37,48,49]. We, however, as detailed in the sterilization process, went through a series of ultrasonication steps prior to cell testing, so we needed to fully characterize what was actually tested with the cells. It is important to point out that the tested samples contained a mixture of dispersed and agglomerated nanoparticles as seen in Figure 4. Based on volume calculations, hydrophilic nanoparticle agglomerates were made of approximately 9 individual nanoparticles, while the hydrophobic nanoparticle agglomerates were made of approximately 31 individual nanoparticles. Surface functionalization with n-octyltriethoxysilane led to increased hydrophobicity of the magnetic iron oxide nanoparticles, as shown in Figure 3, but it also resulted in the formation of significantly larger aggregates as seen in the DLS data. The heterogeneity of particle sizes was difficult to hypothesis test against cytotoxicity since both hydrophilic and hydrophobic nanoparticles were mixtures of agglomerates and dispersed particles. To find a potential reason for our cytotoxicity data difference between PC12 and RVM cells as shown in Figure 5, we looked further into utilizing DLS and ζ-potential measurements. 

The uncoated hydrophilic Fe_3_O_4_ nanoparticles had a ζ-potential measured within previously reported value ranges (−2 to −17 mV) at neutral pH [50,51,52]. The ζ-potential depends heavily on pH and electrolytes in the medium, as well as material properties resulting from synthesis, leading to variations. Based on our ζ-potential measurements, the silica-coated hydrophobic nanoparticles had higher net negative-charge (−27.7 ± 8.2 mV) in aqueous medium compared to the uncoated hydrophilic Fe_3_O_4_ nanoparticles. This is likely due to hydrophilic Fe_3_O_4_ having less deprotonated surface hydroxy (OH) groups compared to the network silica resulting from the n-octyltriethoxysilane reactions. There have been reports that higher negative-charge on nanoparticles affect uptake properties [53]. It is widely agreed that aside from size and surface charge, hydrophobicity plays an important role in the cytotoxicity of nanoparticles. There are conflicting results in the literature regarding hydrophobic coatings and their effects on cytotoxicity. For example, some studies have shown that adding a hydrophobic coating such as oleic acid can reduce the cytotoxicity of nanoparticles [54], while other studies have suggested that cytotoxicity and genotoxicity are induced [26,55]. Thus, modulating the hydrophobicity of the nanoparticles is an important factor for biological applications. In the future, we plan to investigate hydrophilic coatings as a way to reduce aggregation [56]. The changes in biocompatibility as shown in Figure 5 may be due to increased interactions of nanoparticles and/or aggregates from increased associations with the lipid-based cell membrane [57,58]. In this study, the hydrophilic nanoparticles significantly associated less with PC12 but not with RVM cells. One potential explanation for this is the use of non-serum media for the culture of RVM cells, meaning that protein adsorption to nanoparticles will not be an issue. Increasing concentration of serum has been shown to produce protein coronas, which can have significant effects on cell-nanoparticle interactions [59]. The formation of a protein corona in the presence of serum can lead to decreased cytotoxicity [60], and may explain the cytotoxicity differences when comparing PC12 versus the RVM cell lines at equal nanoparticle treatment amounts. Thus, it is possible that differences in cytotoxicity and associations may be due to either cell-type differences, hydrophobicity, or the presence of serum (biomacromolecules).

The major goal of this study was to assess the neurotoxicity of the as-prepared metal oxide nanoparticles. Neurodegenerative diseases are associated with loss of neurons, leading to loss of mental and/or physical functions. Neural stem cells, or progenitor cells which can differentiate into neural-type cells, are under heavy investigation for transplant into the patients to reverse disease progression [61]. One of the most important challenges in stem cell transplantation is real-time monitoring of transplanted cells to assess survival, migration, and differentiation. The nanoparticles in this study were able to label a significant amount of PC12 and RVM cells, even with just one treatment at a low concentration. Additionally, it is important to understand that the differentiation timelines for PC12 and RVM cells to acquire functional neurons are vastly different. PC12 cells are derived from rats, which have a fully developed brain in approximately 15 days [62]. In comparison, human neural stem cells can take several months to reach a fully mature neuronal phenotype [63]. The results presented in this study show a one-week differentiation profile (for each cell type) that can be used as a model for testing the effects during early neuronal differentiation processes. Long-term experiments (e.g., 2–6 weeks) are eventually necessary to conclude if there are any negative effects on neuronal maturation. For future biological characterizations (involving human cell lines such as RVM), utilizing the presented iron oxide nanoparticles should be performed with and without serum to accurately identify nanoparticle interactions to streamline the clinical translation process.

## 5. Conclusions

Magnetite nanoparticles were successfully synthesized using the hydrothermal methods that produced aggregated nanoparticles. The nanomaterials were further surface functionalized with n-octyltriethoxysilane to increase hydrophobicity which resulted in decreased cytotoxic effects. The hydrophobic coating significantly altered the size of the individual nanoparticles as well as the size of the aggregates. It was also important to analyze the iron oxide particle samples before and after the sterilization process. Regarding biological studies, significant differences in toxicological profiles were observed in two mammalian cell lines (that can differentiate into neuronal cells). The hydrophilic nanoparticles had decreased cellular association in PC12 rat pheochromocytoma cells, but not in RVM human neural stem cells, which may be due to serum adsorption. We identified a safe treatment dose in which no significant changes to neurite outgrowth and differentiation profiles were observed in the two tested cell lines. We currently utilize these hydrophobic nanoparticles to engineer hydrophobic microspheres (for tissue engineering) and defining the toxic potential of the incorporating iron oxide species was an important and necessary characterization step with long-term effects in mind for future clinical studies.
